# Novel transportation capsule technology could reduce the exposure risk to SARS-CoV-2 infection among healthcare workers: A feasibility study

**DOI:** 10.1017/ice.2020.352

**Published:** 2020-07-22

**Authors:** Basel Almuabbadi, Huda Mhawish, Bobby Marasigan, Alva Alcazar, Zahraa Alfrdan, Nasir Nasim, Abdulrahman Alharthy, Ziad A. Memish, Dimitrios Karakitsos

**Affiliations:** Critical Care Department, King Saud Medical City, Riyadh, Saudi Arabia

*To the Editor—*Previous studies have documented high infection rates of healthcare workers (HCWs) with severe acute respiratory coronavirus virus 2 (SARS-CoV-2) during the current coronavirus disease 2019 (COVID-19) pandemic.^1–4^ Apart from the routine bedside care, HCWs are exposed to the risk of SARS-CoV-2 cross infection during the transportation of COVID-19 patients. In our healthcare facility, King Saud Medical City (Riyadh, Saudi Arabia), we have employed the recently developed transportation capsule Epi-Shuttle (Epi-guard AS, Torsnesvein, Norway) to reduce the aforementioned risk during the transportation of COVID-19 patients to and from the intensive care unit (ICU). No data exist in the literature regarding this technology. Briefly, the transportation capsule is an innovative, single-patient, isolation system composed of cleanable materials protecting patients from contaminated environments and protecting HCWs from infected patients. The capsule is compatible with standard mechanical ventilation circuits integrating negative- and positive-pressure technology and protective filters against cross contamination by highly infectious pathogens. In the negative-pressure mode, the isolation system protects HCWs from the contaminated air particles shed by infected patients, and in positive-pressure mode, it protects patients from hazardous environmental agents. The recommended disinfection solution for cleaning the capsule after each use is peracetic acid, according to the manufacturer.^5^ We are reporting our preliminary experience using this new technology.

**Fig. 1.**
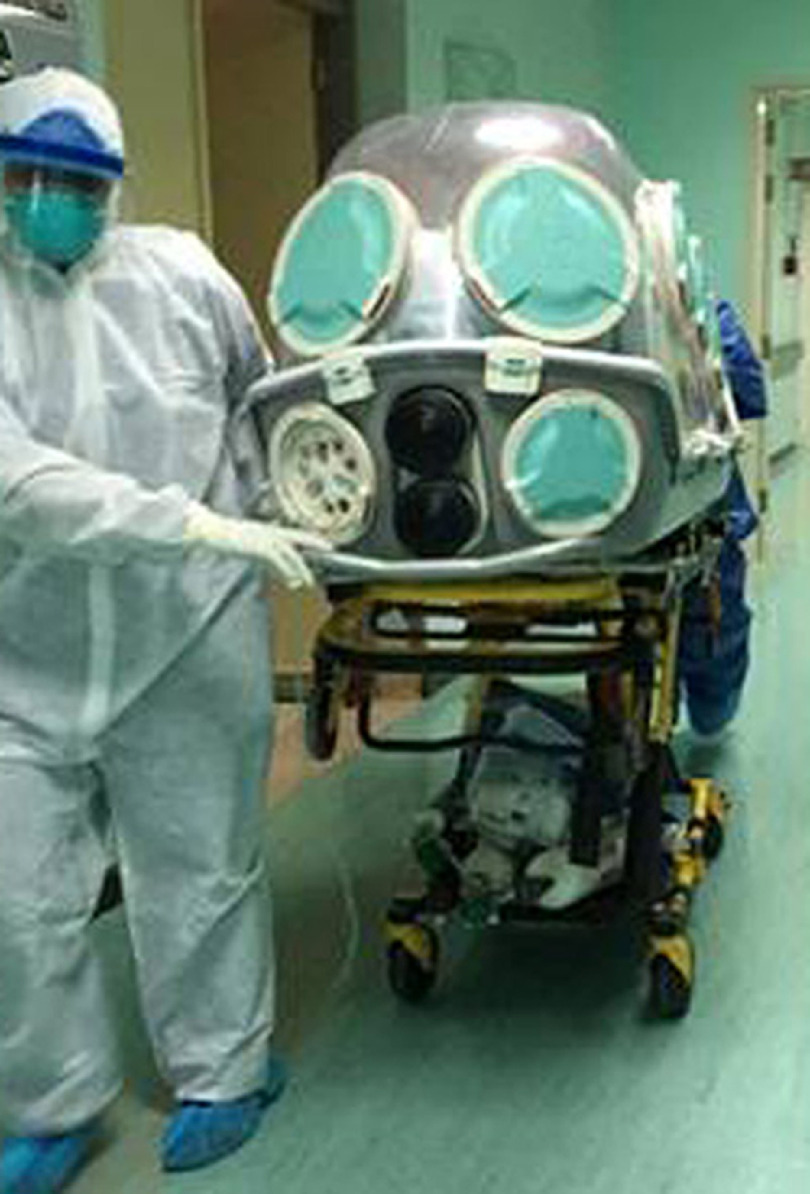
Our healthcare workers transporting a critically ill COVID-19 patient using the insulated capsule.

The ICU of King Saud Medical City (Riyadh, Saudi Arabia) is a 200-bed polyvalent unit designated by the Saudi Ministry of Health (MOH) for the treatment of COVID-19 patients. As of April 2020, 480 patients with COVID-19 had been admitted to our unit, including 380 critically ill patients and 100 patients with moderate-to-serious disease. At that time, we had transported 190 critically ill COVID-19 patients from our healthcare facility to other COVID-19–targeted hospitals according to the Saudi MOH surge plan. The tactical plan has been constantly reviewed and adjusted based on the influx of COVID-19 cases in our ICU. All data were collected and retrospectively analyzed. Of these 190 transported patients, 100 patients had been intubated and 90 patients were on oxygen-supportive therapies. Also, 109 HCWs were employed for the transportation of COVID-19 patients: 39 physicians, 20 respiratory physiotherapists, 20 ICU nurses, and 30 paramedics (Fig. 1). Apart from utilizing the capsule, all HCWs used personal protective equipment according to the World Health Organization recommendations.^5^ None of the 109 HCWs became infected. Moreover, all awake (ie, nonintubated) patients reported a high level of comfort during transportation. Full treatment of ICU patients via access ports, which facilitated emergency procedures (eg, intubation and insertion of central lines) has been possible. In conclusion, the insulated patient capsule has proven to be an efficient technology for the transportation of COVID-19 patients. The capsule has shown good compatibility with ventilator circuits and full treatment of ICU patients as well as ambulance stretchers. Most importantly, none of our HCWs was infected in the transportation process. Large prospective studies are required to confirm or refute the present findings.
